# Characteristics of expert opinions on insanity accepted by NZ Courts

**DOI:** 10.1186/s40352-016-0043-9

**Published:** 2016-12-08

**Authors:** Graham W. Mellsop

**Affiliations:** Department Psychological Medicine, University of Auckland, Auckland, New Zealand

**Keywords:** Legal insanity, Psychiatric expert opinion reports

## Abstract

**Background:**

Health and justice have to communicate whenever the question of legal responsibility is raised with respect to a person accused of a serious crime. Both recommendations and practices on expert report design and content vary widely.

**Methods:**

This paper briefly reviews the characteristics of 27 reports accepted as persuasive in contested New Zealand cases.

**Results and conclusions:**

Relative brevity, presenting the opinions within a court friendly structure, and emphasising the information available around the time of the events, as opposed to information clinically or legally reconstructed, all appear to be important.

## Background

Health and justice have to communicate whenever the question of legal responsibility is formally raised with respect to a person accused of a serious crime. Clinical and legal definitions of insanity do not match up in a 1:1 fashion. Once a criminal charge has been laid, the New Zealand (NZ) Justice system operates on adversarial, as opposed to inquisitorial, principles. The defence can seek a verdict of not guilty by reason of insanity (NGRI). In which case, to assist the Court, both defence and prosecution seek independent psychiatric opinions on the applicability of criteria prescribed in section 23 of the NZ Crimes Act (1961) and subsequent, relevant, case law. From the outset the situation is contested in the sense that the prosecution and the defence seek opposite outcomes. Psychiatric evidence is presented to the court by expert witnesses whose opinions are separately sought by both the prosecution or the defence. Those witnesses are expected to be dispassionate and their opinions uninfluenced by who asked them or who arranges their payment. But only the expert(s) for one side apparently end up being believed.

The NGRI defence is usually only run where a guilty verdict would result in significant imprisonment. As in many jurisdictions the relevant law is similar to the original British M’Naghten rules (Every-Palmer et al. 2014). It essentially requires that *at the time of the act(s)* the accused suffered from a “disease of the mind” of such a degree that either s/he did not know the “nature and quality of their actions” or if they did, they “did not know that their actions were wrong”.

There is a small literature on forensic psychiatric report writing and a variety of alternate templates, schemata, and guidelines. Reputable textbooks generally refer to content issues and the need for impartiality, comprehensibility and demonstrated logic, but offer little guidance on structure (Galpin 2007; Bowden 1990; Freckelton 2007). Bowden (Bowden 1990) and Galpin (2007) refer to standard medical/psychiatric clinical headings and Freckleton (2007) only to principles. In NZ (population 4.5 m; forensic psychiatrists, 45), Allnutt and Chaplow (2000) have argued that psychiatrists should resist giving opinions on the ultimate legal issue even when encouraged to do so by legal participants in the justice system. They also note that it is frequently necessary to obtain collateral information (such as family, development, health and other observations) from third parties and review other sources such as previous psychiatric notes, but they do not refer to using other witness statements or the police interview in the days immediately following the event, despite the Court’s critical interest in the person’s mental state at the time of the alleged offence. Separating the “factual”, which includes the inferred (Galpin), or the “findings” (Allnutt and Chaplow), from the opinion is typically recommended. The Courts decisions have been consistent with the authors opinions as an expert witness in 27 consecutive NZ cases. A brief, qualitative, examination of the characteristics of those reports may inform subsequent practice or textbook descriptions of report requirements in these very important situations.

This brief paper attempts to inform discussion on the characteristics of expert witness reports which are most helpful to the legal processes.

## Method

In the last six years the present writer has prepared 27 reports at the request of either the defence or the prosecution in such contested cases. (Not included are three reports requested by the defence and where the writer opined that the accused was legally sane at the time of the acts as the issue was then never contested).

The 27 will be briefly reviewed in this paper to contribute to the guidance as to report characteristics which appear to influence court decisions.

## The results

Most of the reports were prepared at the request of the Crown (prosecution), but the submitted reports favoured the defence in approximately half of the cases (See Tables 1 and 2). The table records diagnoses, such as schizophrenia [Sc],, Bipolar disorder [B A D], the charges, such as murder or grievous bodily harm [GBH] and minimal prior treatment details such as AP [antipsychotic medication], LAI long acting injections].

**Table 1:** Numbers of reports requested by the defence and prosecution and the numbers which the resultant reports favoured

Opinion		
	Sought by	Favoured
Defence	3	13
Prosecution	24	14

**Table 2:** Summary characteristics of the 27 reports

Age/Sex	Request origin	Crime	Diagnosis	Ethnicity	Report Opinion	Prior Treatment Issues
M 44	Defence	G B H	Sc	Pacific	Insane	medication non-adherence
M 23	Crown	Murder	depr	European	Insane	Treatment non-adherent
M 20	Crown	Murder	Sc	Indian	Insane	Illness not recognised by Psych
M 30	Crown	Arson	Drugs	Indian	Sane	Illicit drugs
M 21	Crown	Rape	B A D	European	Insane	Inadequate medication dose
M 61	Crown	G B H	Sc	Maori	Sane	No treatment
M 40	Crown	Murder	B A D	European	Insane	Low lithium prescribed
M 40	Crown	Murder	Sc	Asian	Insane	Had ceased LAI (depot)
M28	Crown	arson	B A D	Pacific	Sane	Severity not recognised by Psych
M 36	Defence	G B H	Sc	European	Insane	Illness not recognised
M 30	Crown	GBH	Sc	Indian	Sane	Illness not recognised
28	Defence	Sexual assault	Drugs	African	Sane	No illness recognised
M55	Crown	Murder	Depression	European	Sane	Severity of illness not recognised
M 38	Crown	Sexual assault	Drugs	Asian	Sane	Drug use hidden
M17	Crown	Arson	Sc	Maori	Insane	Nil
F24	Crown	Murder	Drugs	Maori	Sane	Drug abuse
M25	Crown	Murder	Sc	European	Insane	Treatment resistant
M40	Crown	Murder	Drugs	Maori	Sane	No treatment
F22	Crown	Attempted Murder	Sc	Maori	Sane	Under treatment
F60	Crown	Murder	Depr	Asian	Sane	Discontinued medication
M32	Crown	G B H	Sc	Maori	Insane	Illness unrecognised
M31	Crown	GBH	Sc	Indian	Sane	Dr ceased AP
M26	Crown	Murder	?ASD	Asian	Sane	Nil
M35	Crown	Murder	Sc	Pacifica	Sane	Non adherence
F39	Crown	Murder	Sc	Indian	Insane	Psychosis unrecognised
M23	Crown	Murder	Sc	Pacifica	Insane	Overt psychosis not treated
M 22	Crown	GBH	Sc	Maori	Insane	Untreated

Footnotes: *Sc* schizophrenia, *ASD* Asperger’s

Where the reports were at the request of the Crown (Prosecutor), but their conclusion favoured the defence argument for NGRI (n = 10), then in subsequent Court proceedings the psychiatric evidence was no longer contested. In two cases, once the defence had access to the Crown psychiatric conclusions rejecting the applicability of section 23 criteria, the insanity defences were withdrawn so those proceedings also became psychiatrically non-contested.

In each of the 27cases the Court’s ultimate decision regarding NGRI was consistent with the writer’s opinion. The following could be seen as characteristics of those 27 reports.

1. Relative brevity: A median length of 8 (maximum 12) quarto sized pages compared with the median of 20 (maximum 60) for the contesting reports.
2. A greater emphasis on evidence available on the day of the event or in the preceding weeks, informing considerations of the presence or absence of psychiatric disorder. By contrast, the contesting reports generally placed greater emphasis on what defendants retrospectively said in the weeks or months following the alleged events -- formal psychiatric interviews for these reports were typically performed 6–12 months after the event.
3. Reports usually did not adopt standard clinical record headings of, for example, history of illness, past history, family history, development, mental state, etc. Rather, they were usually structured around psychiatrically relevant observations under three time specified headings, viz. (i) Leading up to the day of the event; (ii) on the day; (iii) subsequently. The material summarised under those headings was extracted from the available witness statements, health records, Police and clinician interviews.
4. All 27 reports concluded with two sections, Psychiatric Opinion and Section 23 Opinion. Under the former heading the arguments for and against a disease of the mind and other Section 23 criteria were provided. Under the final, Section 23 heading, the writer always gave an opinion based most often on the balance of probabilities, though sometimes to the higher standard of beyond reasonable doubt. In practice, the latter only applied in relation to the presence or absence of the ‘disease of the mind’ component.

## Discussion and conclusions

Twenty seven cases is a small sample for most quantitative research so this work needs to be considered qualitative, except for the fact that all the judicial decisions were consistent with the concluding opinions expressed in the writer’s reports. The work was completed in small country, but it is clear that the New Zealand NGRI concept is very similar to that in the jurisdictions of many other countries (Every-Palmer et al. 2014; Mellsop et al. 2016). Those publications examine the many similarities and practical differences in how psychotic accused persons can utilise a legal insanity defence in many major countries of the Pacific Rim.

Psychiatrists have traditionally placed credence on information obtained from their own interviews. Although in theory they recognise the temporal instability of evidence, particularly where an interviewee has had endless “recall” sessions with relatives, legal counsel, and/or their treating team. Particularly in the forensic field there is a necessity to be watchful for contamination by the rarer malingering, false memories or straightforward dishonesty. The emphasis in the 27 reviewed reports on the greater value of antecedent information, and that obtained on the actual day or subsequent police interview, may have appealed to a more cynical legal or judicial logic. To study this by having an open and independent examination of a series of reports from both prosecution and defence expert witnesses would possibly provide more reliable information, but the practicality of such a project is at best daunting.

Even the writer’s relative brevity results in longer reports than one UK recommendation of 2–3 pages (Faulk 1988).

In contrast to the Allnutt and Chaplow view (Allnutt & Chaplow 2000), it can be inferred that NZ Courts have no problem receiving clear psychiatric opinions on Section 23 criteria, and do not regard this as pre-empting their own powers.
